# Editorial Department

**Published:** 1855-01

**Authors:** 


The American Journal of Medical Sciences for October, has a review of the trial of John Hendrickson, Jr., for the murder of his wife, at Albany, in 1853. We find the initials C. A. L. at its conclusion, and it bears also the stamp of the logical mind of our friend Prof. Charles A. Lee.

We would gladly publish the whole of this very able review but must limit ourselves to such extracts from it as our space admits.

"A brief history of the case will be necessary in order to appreciate the nature of the testimony, on which we design to make a few comments.

"The accused, John Hendrickson, Jr., a young man, twenty years of age, born of respectable parents, married, at the age of eighteen, Maria Van Dusen, a young lady of seventeen, also of highly respectable connections, well educated, amiable, and intelligent. There is no evidence that they lived together unhappily; and although the District Attorney, in his opening speech, spoke of the prisoner having communicated syphilis to his wife, there is no satisfactory proof that such had been the case; but, on the contrary, it is quite evident that she had labored under a severe form of leucorrhœa. On the night of the 7th of March, 1853, after attending church in the evening, she retired to bed with her husband at her father-in-law's, between ten and eleven o'clock, complaining of a severe pain in her head, hips, and loins, and at two o'clock was found dead by her husband, 'occupying nearly the center of the bed, lying at full length on her back, with her hands either crossed, or lying down by her side, the bedclothes covering her person.' A coroner's inquest was held the same evening, and a *post-mortem* examination ordered, which was made thirty-six hours after death, by Dr. J. Swinburne, Dr. Ingraham, and the coroner, Mr. Smith, being present. Four days after, the the body was disinterred, and further *post-mortem* dissection made, the first, for some reason not specified, having been incomplete. The principal appearances noticed are the following: Great pallor of the surface; calm expression, and no distortion of features; sugillation on the posterior parts of the body; great rigidity and *elasticity* of the muscles; lungs and heart healthy; heart empty of blood, except a small clot in the right auricle; the vena cavas partly full of dark fluid blood; stomach and small intestines empty; liver healthy; gall bladder half full of bile; mucous coat of stomach very red, lined with a thick reddish mucus, corrugated, the stomach itself contracted to two inches in diameter, and its coats hypertrophied; the mucous coat of duodenum also corrugated, and more congested than that of the stomach; uterus enlarged to twice its natural size, hardened, and its cervix slightly ulcerated, with adhesions to the rectum and small intestines; ovaries considerably enlarged; spleen and pancreas healthy; urinary bladder contracted; brain healthy, no congestion; tongue white, and a little swollen; a small ecchymosed mark on inside of lower lip, showing a cut about a quarter of an inch in length (throat, œsophagus, spinal cord, and lower portion of intestinal canal not examined.) At the next examination, five days after, the kidneys were found healthy, feces in the cæcum; — portions of lung, liver, and pancreas, with four ounces of blood from cavity of chest, were removed, placed in jars, and carefully covered.

"To the question, on the subsequent trial, 'What was the cause of death?' Dr. S. replied:

'Acrid poison. I base it on this: I find entire emptiness of the stomach and small intestines, so far as fecal matter is concerned; also contraction and corrugation of the same to a great extent. I find in place of that a reddish *viscid* mucus adhering to the coat of the stomach and intestines; the emptied condition of the gall-bladder; the appearance of the tongue. *I inferred from these that vomiting had taken place,* and that, too, induced by some acrid matter, which would not only expel the contents of the stomach, but of the small intestines, the presence of which acrid matter would induce the vomiting.'

"To the question, 'What would induce your belief that she vomited?' the witness replied:

'I believe the act of vomiting is accompanied by more or less contraction of the stomach; where that act is induced by the presence of acrid matter, *the contraction will be proportional to the material used, be it more or less irritating.*' 'The corrugation would be owing, in part, to the contraction of the muscles, and part to the irritating matter applied to the mucous surface.' 'The rigidity of the body presented the appearance of a person destroyed by anything *which would produce a sudden spasm or contraction of the muscles.* The appearance of the stomach and intestines proved to me *conclusively* that she had vomited, and to such a degree as could not be produced by ordinary causes; and I think the effort at vomiting continued until exhaustion took place. My reasons are these: *the blood, in the first place, was thrown from the center to the surface; also, the extreme pallor of the countenanee,* which always attends exhaustion from vomiting.'

"To the question, 'Will you state, in your judgment, from what poison she came to her death?' Dr. Swinburne replied:

'*I supposed the deceased died from aconite, from the fact of the appearance on the post-mortem examination being so identical with those of the dogs and cats,*' (experimented on by Dr. Salisbury with tincture of aconite.)

"The counsel then asked: 'What is the strength of your opinion, that she died of poison?' Dr. S. replied:

'*I have no doubt of it; I have no doubt that she vomited; one of my reasons for thinking she died of poison, was her having vomited, and also the absence of congestion. Had Mrs. H. died of natural causes, more or less natural contents would have been found in the small intestines,* and they would have presented a healthy appearance, relaxed instead of contracted; also, *the circulating system would have presented a very different appearance.* In all forms of death by asphyxia, syncope, apoplexy, epilepsy, and all the other forms of convulsive diseases, you have fullness or engorgement of the heart and all the important viscera.' Again: 'From my reading, knowledge, and experience, I am prepared to express an opinion as to what caused the morbid condition of the stomach and bowels—*it was aconite, or the principle of aconite.* Had the poisonous matter passed off with any of the fecal matter, I think the *rectum* would have presented the same appearance as the intestines, only in a less degree; can assign no possible way, except by vomiting, how the fecal matter could have been removed.

'What external appearances would you look for, where death has been by aconite, in three or four hours?'

*'Should expect to find rigidity of all the voluntary muscles; also, extreme pallor from vomiting.'* "

Prof. Lee then proceeds to discuss the real value of the redness of the stomach as an evidence of acrid poisoning, and shows, conclusively, by all the most competent authorities, that is a most uncertain diagnostic, being found in many cases as a purely *post-mortem* appearance.

"It is very evident, then, that redness, quite equal in degree to that observed in the case of Mrs. Hendrickson, is no uncommon appearance in *post-mortem* examinations, and the redness would seem to be intense in proportion as the death has been sudden and the circulation active. The redness in the present instance was no greater than is usually met with in cases where death is as sudden. The 'reddish mucus' in the stomach may, perhaps, be satisfactorily accounted for from the presence of the coloring matter of tomatoes, which the deceased ate freely shortly before her death. The microscope might have settled this question definitely.

"The other phenomena mentioned, on which considerable stress appears to have been laid, viz: pallor of the surface, and rigidity, and elasticity of the muscles, &c., are even less significant or characteristic of poisoning than the slight congestion already noticed. Neither can be considered as indicative of modes of death; their absence, indeed, might be worthy of note in a suspected case, but not their presence. In all cases of death from irritant poisons, especially the narcotico-irritants, which we have had an opportunity of observing, there have been crimson or livid-colored patches on various parts of the body and considerable tympanitis, though we cannot affirm that these phenomena are invariably present in such cases. There has also been considerable bloody, frothy mucus in the mouth, fauces, and œsophagus, especially when vomiting has been severe. No such phenomena were noticed in the present instance; only we are told that the tongue was 'swollen, and very white,' an appearance not particularly indicative of gastric irritation. It is also worthy of note, that the 'pallor' is attributed to the 'blood having been thrown from the center to the circumference, by vomiting,' which we should suppose would have the opposite effect.

'The rigidity of the body,' says Dr. S., 'presented the appearance of a person destroyed by anything which would produce a sudden spasm or contraction of the muscles.'

"Here it is assumed that the deceased died from spasm, and that the spasmodic state of the muscles continued after vitality was extinguished (thirty-six hours after death). No allusion is made to the *rigor mortis*, as a common cadaveric phenomenon; but it is claimed throughout the direct testimony, that the stiffness of the body was owing to some poison which had caused severe spasm; and because the witness had noticed the same rigidity in dogs, destroyed by aconite, he did not hesitate to express the opinion that the deceased came to her death from the same poison. Now cadaveric rigidity takes place in all classes of animals alike; coming on as soon as muscular irritability ceases, confined to the muscular system in many cases, giving extreme rigidity to the body, its degree and duration, *cæteris paribus*, being directly as the muscular development; continuing longer the later it occurs, and *vice versâ;* influenced greatly by the nature of the disease, or the cause of death; appearing more speedily, and lasting a much shorter time, when death has occurred from some chronic wasting disease, as phthisis, fever, scurvy, &c., coming on slowly and being strongly developed, and lasting often for several days, when the death has been occasioned by acute inflammation of the stomach, or by irritant poisons. We have known cadaveric rigidity continue four or five days after death from cholera. Any one conversant with these facts, would hardly dare derive any positive conclusion as to the cause of death from the presence of muscular rigidity. We dismiss it, therefore, with the 'pallor' and 'congestion,' as wholly insufficient to justify the conclusions deduced from it.

"Considerable stress is also laid by the witnesses, on the 'corrugation and contraction of the stomach;' but these are so often met with in death from natural causes, that no significance can be properly attached to them. Prof. Horner has shown that *rugæ* of the stomach are quite common in *post-mortem* examinations, and that they appear in cases where no stimulants or irritants have been applied, and in stomachs perfectly healthy, and that they are more frequently met with in cases of sudden death; and yet it is assumed, in the case of Mrs. H., that these rugæ were owing to the *astringent* properties of aconite, which has heretofore been regarded as a *paralysant*, and not a stimulant." * * * *

"The diminished calibre of the stomach and intestinal canal, was in all probability the result of sudden death, leaving the organic contractility of the muscular fibre unimpaired.

"Extensive experiments have been made with aconite upon the lower animals; but corrugation of the stomach has never been claimed as one of its *specific* effects. It was never observed by Orfila, or by Fleming."

The chemical analysis of the contents of the stomach was made by Dr. Salisbury, of Albany. We extract the following summary of his process:

"In examining the chemical evidence, we must first express our regret that the details of the processes employed by Dr. Salisbury, are so imperfectly given. We can only judge of the correctness of the results from the very meagre account presented in the evidence as published. We shall, however, if we mistake not, discover such errors in the proceedings as to vitiate in a great degree, if not wholly, the conclusions which are drawn from them. We are first given to understand, that with 'a small portion of the mucous surface of the stomach and duodenum,' Dr. S. first tested for *prussic acid*, then for *some* of the *mineral poisons* (arsenic, corrosive sublimate, the antimonial compounds, the mineral acids, muriatic, nitric, and sulphuric), then oxalic acid; also for *morphine, strychnine, stramonine,* (daturia?) and also 'for other poisons,' but without success. These various processes, we presume, must have taken a considerable portion of the 'small portion of mucous membrane' employed. We have no means of judging of the accuracy of the tests employed, for few, if any, details whatever are given.

"Dr. S. then states, 'that he *tested* for *aconite*, and found it by proceeding as follows: He took, as before, a small portion of stomach and duodenum, digested it in alcohol over a water-bath, then filtered, evaporated the filtrate partially; the oily matter rose to the surface; this he separated by decantation, and then absorbed it from the surface by bibulous paper; then mixed the solution with purified animal charcoal, agitating for some time after mixing, filtered, and applied his acid tests as follows: First, he boiled a small portion of the solution with sulphuric acid; the solution was turned a *deep port-wine red color;* then the same process with hydrochloric acid gave a *light port-wine red color;* then with nitric acid, and the *solution remained clear;* and from these tests, Dr. S. says, '*he inferred the presence of aconitine*,' but on what grounds we are totally at a loss to discover. *Sulphuric acid* boiled with aconitine gives *a dark brown tint*, according to Taylor, instead of a *port-wine red color;* and animal matters, boiled with sulphuric acid, will give the latter color *without the presence of aconitine;* so say Taylor and the best authorities on legal medicines. (*Taylor on Poisons*, Am. Ed. p. 615.)

"Prof. Emmons, in a published letter to Gov. Seymour, of New York, states as the result of his experiments, that 'sulphuric acid boiled with the tincture of aconite, obtained from the same sample as that supposed to have been sold to Hendrickson, *lost most of its red color*, and became quite pale; boiled with pure aconite, the s lution remained colorless, and the same result took place when boiled with nitric acid;' but that when he added oil or animal matters to the mixture of aconite, then he 'obtained the red colors spoken of by Dr. Salisbury, and the same also occurred *when he employed the same tests on these animal matters alone*.'

"We think there can, therefore, be no doubt whatever that the results arrived at by Dr. S. were entirely due to the presence of organic matters, and not to aconitine, although the color obtained by Dr. Emmons with sulphuric acid differs from that laid down by Taylor as resulting from a solution of pure aconitine.

"But we are not called upon to reconcile this discrepancy; our aim is merely to show the incorrectness of the inference that aconitine was discovered by the process employed by Dr. S."

Prof. Lee concludes the review with the succeeding remarks:

"We have not designed to offer any comments upon the *moral* evidence submitted in this case—any further, at least, than it is connected with the medical and legal evidence. We may, however, be allowed to say, that it lends little or no confirmation to the belief that poison was administered to Mrs. H. It was not proved that the prisoner ever purchased aconite, knew of its properties, or had it in his possession. The charges brought against his moral character, in the opening speech of the prosecuting attorney, particularly in regard to his having had gonorrhœa, and having imparted the same to his wife, were wholly unsustained by any evidence offered on the trial. The object aimed at, viz: prejudicing the minds of the jury against the prisoner, was, however, perhaps as fully attained as if the charges had been proved. There was no evidence that any vomiting had taken place; indeed, everything went to show that it had not; the countenance of the deceased was mild and placid; no distortion, no dishevelling of the hair, no wrinkle or spots on her clothes, no signs of violence upon the exterior of the body, no marks of suffocation; the deceased lay as if asleep, and everything in the room in the same condition as when she retired to rest. This does not look like severe vomiting, or death from violence—unless, possibly, from chloroform—but this is not charged. The Judge (Marvin,) in his charge to the jury, seems to take the ground that, if the medical witnesses, called for the defence, could not prove what was the actual cause of death, it was right to assume that the deceased came to her death by violence on the part of her husband! 'It would be more satisfactory,' he says, 'if these medical witnesses had assigned some natural cause for her death;' as if the burden of proof rested on the prisoner, instead of the people, although he expressly disclaims any such ground. We confess that the case is enveloped in considerable mystery, but no more than enshrouds hundreds of cases of sudden death. We were called once to see a lady who, in the enjoyment of a comfortable state of health, while attending a social gathering at her own house, was seized with a fainting fit, and died in spite of all the means employed before our arrival. We made a careful *post-mortem* the next day of all the vital organs, but there was no cause of death discoverable. The walls of the heart were thinner than usual, and syncope was occasioned by violent mental emotion, which resulted fatally. We have known several cases of sudden death from syncope, without any *post-mortem* lesion sufficient to account for the death. Fatal cardiac asthenia may be produced by mental emotions, and no *post-mortem* change or lesion discovered. It may have been so in the present instance. There was considerable *uterine* disease, as ulceration and congestion of the os uteri, and leucorrhœa; sympathetic disturbance of the heart may have been occasioned by mental emotion acting upon this predisposition, and death occurred as in the case already alluded to. Or, she may have inhaled chloroform to relieve pain, of which she had complained much during the day; or, she may have taken an overdose of *homœopathic* pills of aconite, of which she had a considerable quantity on hand just before her death, and which she carried in her pocket (of which none were found afterwards.) Many causes might be assigned to account for her death, any one of which would be more probable than that she was poisoned by an ounce of tincture of aconite. There is one fact stated by the Judge in his charge, as of very great importance, and one which we have not as yet particularly noticed, and that is, a small *ecchymosis* or bruise on the inside of the lip. The Judge says this 'furnishes overwhelming evidence of guilt to his mind.' Dr. Swinburne describes it in his testimony as 'a black and blue bruise between the size of a sixpence and a ten-cent piece, inside of the lower lip, and a little to one side; in it there was a cut about a quarter of an inch long; it must have occurred before death.' This slight mark is exaggerated into a degree of importance it does not deserve. It was proved by the mother that the deceased complained two days before her death of a sore on the same lip. The evidence that the ecchymosis was caused during life is by no means satisfactory; indeed, no evidence is gone into, or facts detailed, which have any bearing on this point. It is stated by the mother, that violent attempts were made by her to open the mouth, for the purpose of administering a little camphor and water, as soon as it was discovered that her daughter was dead; and probably this was not long after she ceased to breathe. The ecchymosis may have been thus produced, though we think it more probable that it existed before death. Such marks, moreover, are not unfrequently caused by the teeth during a convulsive paroxysm. Standing alone, it by no means proves any violence on the part of the accused.

"Another feature in this trial is worthy of mention, perhaps, and that is the great importance attached to the confident and positive statements of Drs. Swinburne and Salisbury, both young men, and comparatively inexperienced, and the little weight which seems to have been allowed to the more careful and judicious testimony of Drs. Emmons and Staats, men of age, professional skill, and enlarged experience. The *positive* statements of the former would probably, with such a court and jury, have outweighed the negative testimony of all the first pathologists and chemists of the age. The result shows very clearly the importance of qualifying and guarding our statements and opinions where facts will allow of another interpretation, and where they fall short of actual demonstration.

"We have thus successively passed in review the most important features in this interesting trial, and made such comments as seemed to us appropriate to the occasion. We know none of the parties, nor are we influenced by any personal considerations whatever. Medico-legal science demands thus much, at least, at our hands. If we have spoken with undue severity, in regard to any testimony offered, it will ever be to us a source of regret. But the interests of science and humanity require that the medical and chemical evidence, on the strength of which the life of a human being has been taken, should be closely scrutinized, and in all the lights which observation, reason, common sense, and true science may furnish us. The case will stand as a precedent in medico-legal questions that may hereafter come before our courts. It is all-important to know whether the questions it involves have been decided correctly or not; whether it may be referred to as a safe or an unsafe precedent for witnesses, courts, and juries in future times. We have sufficiently indicated our opinion. We are ready, however, to retract whenever our reason is convinced that we have been led into error—not before. It is in vain, for it is too late, to lament the probable sacrifice of an innocent man; but, if what we have written serves but to throw an additional safeguard hereafter around the wrongfully accused, our purpose will have been fully accomplished. We will venture one remark more. It seems to us no less strange than lamentable, that in a case which appears to have rested solely on anatomical, or pathological and chemical evidence, the counter opinions and statements of the leading men in these departments of science in the country should have had so little weight with the executive or judicial power as not to lead to a commutation of punishment, nor even an order for a new trial, which was demanded. We pretend not to fathom the hearts or the motives of men, but we think we see in the present instance an example and illustration of the force of outward pressure—of popular excitement and prejudice—on witnesses, counsel, judges, and jury, which makes us question, at times, whether the boasted right and privilege of trial by jury, be, indeed, a blessing or a curse. C. A. L."

